# Impaired olfaction is associated with cognitive decline and neurodegeneration in the brain

**DOI:** 10.1212/WNL.0000000000006919

**Published:** 2019-02-12

**Authors:** Christina S. Dintica, Anna Marseglia, Debora Rizzuto, Rui Wang, Janina Seubert, Konstantinos Arfanakis, David A. Bennett, Weili Xu

**Affiliations:** From the Aging Research Center (C.S.D., A.M., D.R., R.W., J.S., W.X.), Department of Neurobiology, Care Sciences and Society, Karolinska Institutet and Stockholm University; Department of Clinical Neuroscience (J.S.), Psychology Division, Karolinska Institutet, Stockholm, Sweden; Department of Biomedical Engineering (K.A.), Illinois Institute of Technology, Chicago; Rush Alzheimer's Disease Center (K.A., D.A.B.), and Department of Neurological Sciences (D.A.B.), Rush University Medical Center, Chicago, IL; and Department of Epidemiology and Biostatistics (W.X.), School of Public Health, Tianjin Medical University, China.

## Abstract

**Objective:**

We aimed to examine whether impaired olfaction is associated with cognitive decline and indicators of neurodegeneration in the brain of dementia-free older adults.

**Methods:**

Within the Rush Memory and Aging Project, 380 dementia-free participants (mean age = 78 years) were followed for up to 15 years, and underwent MRI scans. Olfactory function was assessed using the Brief Smell Identification Test (B-SIT) at baseline, and categorized as anosmia (B-SIT <6), hyposmia (B-SIT 6–10 in men and 6–10.25 in women), and normal (B-SIT 10.25–12 in men and 10.5–12 in women). Cognitive function was annually assessed with a battery of 21 tests, from which composite scores were derived. Structural total and regional brain volumes were estimated. Data were analyzed using linear regression and mixed-effects models.

**Results:**

At study entry, 138 (36.3%) had normal olfactory function, 213 (56.1%) had hyposmia, and 29 (7.6%) had anosmia. In multiadjusted mixed-effects models, hyposmia (β = −0.03, 95% confidence interval [CI] −0.05 to −0.02) and anosmia (β = −0.13, 95% CI −0.16 to −0.09) were associated with faster rate of cognitive decline compared to normal olfaction. On MRI, impaired olfaction (hyposmia or anosmia) was related to smaller volumes of the hippocampus (β = −0.19, 95% CI −0.33 to −0.05), and in the entorhinal (β = −0.16, 95% CI −0.24 to −0.08), fusiform (β = −0.45, 95% CI −0.78 to −0.14), and middle temporal (β = −0.38, 95% CI −0.72 to −0.01) cortices.

**Conclusion:**

Impaired olfaction predicts faster cognitive decline and might indicate neurodegeneration in the brain among dementia-free older adults.

At present, 46.8 million people have dementia worldwide. This number is expected to reach 115.4 million by 2050.^1^ It is therefore important to identify possible risk factors and predictors to improve the early detection of those at high risk of dementia.

The prevalence of olfactory impairment in the general population is approximately 3.8% to 5.8%, with proportions increasing to 13.9% in individuals older than 65 years of age.^2^ In recent years, several studies have shown an association between impaired olfactory function and risk of cognitive impairment^3,4^ and Alzheimer disease (AD) dementia.^5,6^ A few longitudinal population-based studies have shown that poorer olfactory performance is associated with cognitive decline,^7–11^ while others have not shown such associations.^12^ In addition, the possible/potential mechanisms that underlie the association of olfactory function with cognitive impairment, AD dementia, and its pathologies are still unknown. One neuroimaging study showed that lower olfactory function was associated with smaller hippocampal volume in patients with mild cognitive impairment and AD dementia, but not in cognitively normal individuals.^13^ Furthermore, cross-sectional associations between olfactory impairment and AD biomarkers (i.e., AD signature cortical thickness, hippocampal volume, and amyloid burden) have also been reported in cognitively normal older adults.^14,15^ To date, questions remain about whether olfactory function is associated with cognitive decline and AD- and non-AD-specific degenerative markers on MRI, which may underlie the olfactory function–cognitive decline association.

In this study, we extended our earlier work by including a longer follow-up time (up to 15 years of cognitive testing) and adding structural imaging data. Using data from a long-term study of dementia-free older adults, we aimed to (1) examine the longitudinal association between olfactory impairment and cognitive decline and (2) explore the association between olfactory impairment and neurodegenerative markers assessed with structural MRI.

## Methods

### Study population

The Rush Memory and Aging Project (MAP) is an ongoing prospective study that investigates risk factors for common chronic neurodegenerative conditions in older adults.^16^ Details regarding the MAP study design and the evaluation protocol have been provided previously.^16^ In brief, participants were recruited from the greater Chicago area from church groups, senior citizen housing facilities, and retirement communities. At study entry and thereafter, all participants underwent a comprehensive clinical assessment, including neurologic examination, medical history, extensive cognitive function testing, and odor identification testing.^16^

**Figure 1:**
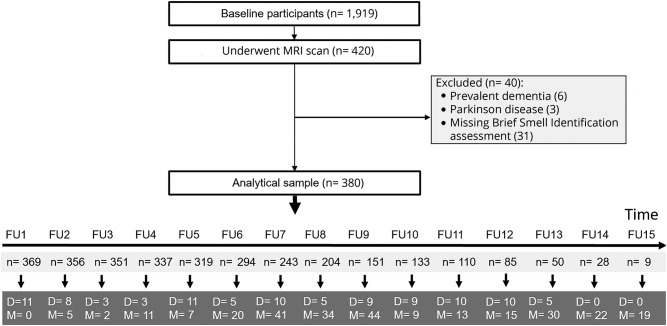
Flowchart of study participants D = died; FU = follow-up; M = missing.

Beginning in 1997 through 2014, a total of 1,919 participants were enrolled. Starting in 2009, participants were invited to undergo an MRI scan. Of the 1,919 enrolled participants, we limited our study sample to the 420 who had undergone a structural brain MRI scan. The study participants were annually followed up for a maximum of 15 years. We excluded 40 participants with prevalent dementia (n = 6), Parkinson disease (n = 3), and missing Brief Smell Identification Test (B-SIT) scores at baseline (n = 31); therefore, the sample for this study was 380 participants (figure 1). During the study period, 99 participants died, and the participation rate of survivors exceeded 90%.

### Data collection

All participants underwent a uniform evaluation with trained staff that included structured interviews, clinical and neurologic examinations, and cognitive testing, which has been fully described.^16^ Data on sociodemographic characteristics (i.e., age, sex, and education), lifestyle factors (i.e., smoking and alcohol consumption), anthropometrics (i.e., body weight and height), medical conditions, and cognitive function were collected at each wave.

Education was recorded as maximum years of formal schooling. Smoking was categorized as never smoked, former smoker, and current smoker. Alcohol consumption was categorized into no/occasional drinking vs light to heavy drinking. Grams of alcohol per day at baseline was a measure of how much alcohol (beer, wine, and liquor) a participant consumed in the past 12 months and was categorized as: less than 1 drink per month to 1 drink per week; 2–4 drinks per week to 1 drink per day; and 2–3 drinks per day to 5–6 drinks per day. Body mass index was calculated as weight in kilograms divided by height in meters squared.

Information on medical conditions including cardiovascular conditions (heart disease and hypertension), diabetes, and stroke was collected based on self-report during the interview and clinical/neurologic examination at baseline. Activities of daily living were assessed at baseline and dichotomized as dependent or not. Blood samples were taken at study entry and the *APOE* gene was genotyped utilizing high-throughput sequencing and dichotomized as any ε4 carriers or ε4 noncarriers.

### Standard protocol approvals, registrations, and participant consents

The study was approved by the institutional review board of Rush University Medical Center and was performed in accordance with the ethical standards laid down in the 1964 Declaration of Helsinki and its later amendments. Informed consent was obtained from all participants. Participants also signed a repository consent that allowed their data to be shared. More information on obtaining data can be found on the Rush Alzheimer's Disease Center Resource Sharing Hub at radc.rush.edu.

### Assessment of odor identification

At baseline, the B-SIT (Sensonics International, Haddon Heights, NJ) was administered. The B-SIT is a standardized, 12-item, 4-alternative forced-choice measure. In this procedure, a booklet is presented to the participant, where each page contains a scratchable patch of microencapsulated odorant. For each item, the examiner scratched the odor patch with a pencil to release the odorant. The patch was then placed under the participant's nose, and the participant was asked which of 4 specific odors the item most closely resembled. The score was the number of correctly recognized odors and ranged from 0 to 12. If responses to 1 or 2 items were missing, each was given a score of 0.25.^17^ If responses to 3 or more items were missing, data on the test were considered missing. The content of the B-SIT is internally consistent, and its scores are in agreement with scores on the 40-item University of Pennsylvania Smell Identification Test, from which it was derived.^17^ Olfaction categories were classified by B-SIT score: anosmia (score <6), hyposmia (men 6–10, women 6–10.25), and normal olfaction (men 10.25–12, women 10.5–12).^18^

### Cognitive function assessment and dementia diagnosis

Cognitive function was assessed at study entry and annual follow-up examinations, with a battery of 21 performance tests in an approximately 1-hour session (see Wilson et al.,^17^ 2006, for details on cognitive assessment). Briefly, *episodic memory* was tested using the immediate and delayed recall of the East Boston Story, Story A from Logical Memory and Consortium to Establish a Registry for Alzheimer's Disease Word List Memory, Recall, and Recognition. *Visuospatial ability* was assessed with a 17-item version of Standard Progressive Matrices and a 15-item version of Judgment of Line Orientation. *Perceptual speed* was tested using Number Comparison, the Stroop Test, and the oral version of the Symbol Digit Modalities. *Semantic memory* was assessed by a 15-item version of Extended Range Vocabulary, a 20-item reading recognition test from the National Adult Reading Test, a 20-item version of the Boston Naming Test, and Verbal Fluency test. *Working memory* was tested using Digit Ordering, Digit Span Backward, and Digit Span Forward. The scores on each test were converted to *z* scores (based on all MAP participants at baseline).^17^ The *z* scores from component tests were averaged to yield a composite score for global cognition as reported in detail in a previous study.^17^

Dementia was diagnosed following the criteria of the joint working group of the National Institute of Neurological and Communicative Disorders and Stroke and the Alzheimer's Disease and Related Disorders Association.^19^

### Brain MRI acquisition

High-resolution T1-weighted anatomical data were obtained on a 1.5-tesla GE (General Electric, Waukesha, WI) MRI scanner, using a 3-dimensional inversion recovery prepared fast spoiled gradient recalled sequence with the following parameters: echo time = 2.8 milliseconds (ms), repetition time = 6.3 ms, preparation time = 1,000 ms, flip angle 8°, field of view 24 × 24 cm, 160 slices, 1-mm slice thickness, 224 × 192 image matrix reconstructed to 256 × 256, 2 repetitions.^20^ We used FreeSurfer (v.5.0) to automatically segment the MRI data. When necessary, manual intervention was used to increase the accuracy of labeling. Whole-brain gray matter volume as well as the volumes of cortical and subcortical gray matter structures were obtained.^21,22^ Total volumes (adding left and right sides) were calculated and converted from cubic millimeters to tenths of percentage of intracranial volume (using the estimate of intracranial volume from FreeSurfer v.5.0). There were 19 participants who were scanned at a different site; therefore, site of scanning as a covariate was controlled for in all analyses.

### Statistical analysis

The characteristics of the study population by the 3 olfaction groups (normal olfaction, hyposmia, and anosmia) were compared using χ^2^ tests for categorical variables and 1-way analyses of variance with Bonferroni post hoc comparisons for continuous variables.

For the longitudinal data analysis, linear mixed-effects models were used to characterize individual trajectories of change in cognition in relation to baseline olfactory function (continuous B-SIT and categorical variables) with baseline global cognition and annual rate of change. The fixed effects included baseline olfaction category (normal olfaction vs hyposmia or anosmia), linear yearly follow-up time, and their interaction (olfaction category × time). To allow for individual differences at baseline and across time, we included random effects for the intercept and slope for time. We controlled for age, sex, education, and *APOE* ε4 in the multiadjusted mixed-effects models as potential confounders. In addition, to account for the possibility of practice effects for the cognitive testing, we included a time-varying retest covariate (“First cognitive assessment” vs “Follow-up assessment”).

For the MRI data analysis, separate linear regression models were used to estimate the relationship between olfactory function and regional brain volumes. The volumes included in the analysis were of the primary olfactory cortex (e.g., amygdala, entorhinal cortex), the secondary olfactory regions (e.g., hippocampus, hypothalamus, thalamus, insula, orbitofrontal cortex),^23^ as well as AD signature areas (parahippocampal, entorhinal, inferior temporal, middle temporal, and fusiform cortices).^24^ In multi-adjusted regression models, we controlled for age, sex, education, APOE ε4, scanning site, and baseline global cognition as potential confounders. To further explore the role of *APOE* ε4 in the examined associations, first stratified analysis by *APOE* ε4 was performed, and then an interaction term between olfaction categories and *APOE* ε4 status was included in the models.

To broadly investigate the associations between odor identification and volumetric markers of neurodegeneration, multiple comparisons were not mathematically corrected for^25^ in order to reduce the chance of type II error. Associations were considered significant at *p* < 0.05, and all statistical analyses were performed using Stata SE 15.0 for Windows (StataCorp LLC, College Station, TX).

### Data availability

All data included in these analyses are available via the Rush Alzheimer's Disease Center Research Resource Sharing Hub, which can be found at radc.rush.edu. It has descriptions of the studies and variables and a dynamic query function to aid searches for data and biospecimens for selected data. There is a login, after which any qualified investigator can submit a resource request.

## Results

Among the 380 participants (mean age = 78 ± 7 years; 76% female), 138 (36.3%) had normal olfactory function, 213 (56.1%) had hyposmia, and 29 (7.6%) had anosmia. Participants with impaired odor identification (hyposmia or anosmia) were older (*p* = 0.001) and had lower global cognitive function (*p* < 0.000). There were no significant differences in education, vascular risk factors (smoking, alcohol consumption, and body mass index), vascular diseases (hypertension, stroke, and heart disease), diabetes, *APOE* ε4, and dependency (table 1). The mean number of follow-up assessments was 9 (±3.4) with a range from 1 to 15. The participants with 9 or more follow-ups (n = 198, 52%) were younger and had a higher global cognitive score at baseline compared to those with less than 9 follow-up assessments (n = 182, 48%). The median time between the baseline assessment and the MRI scan was 2 years (interquartile range = 6). Participants with incomplete or missing odor identification scores did not differ significantly from those with complete data in population characteristics.

**Table 1:**
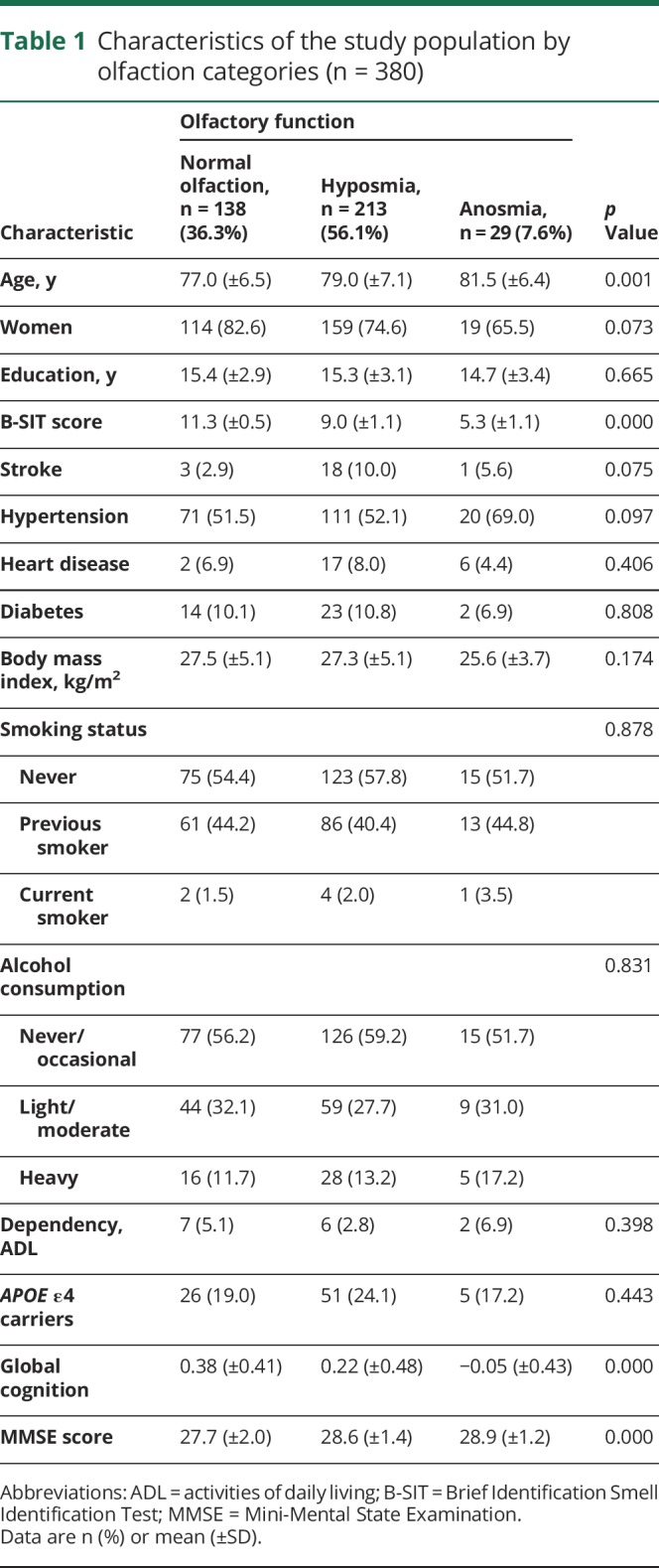
Characteristics of the study population by olfaction categories (n = 380)

### Relationship between olfactory function and cognitive decline

**Figure 2:**
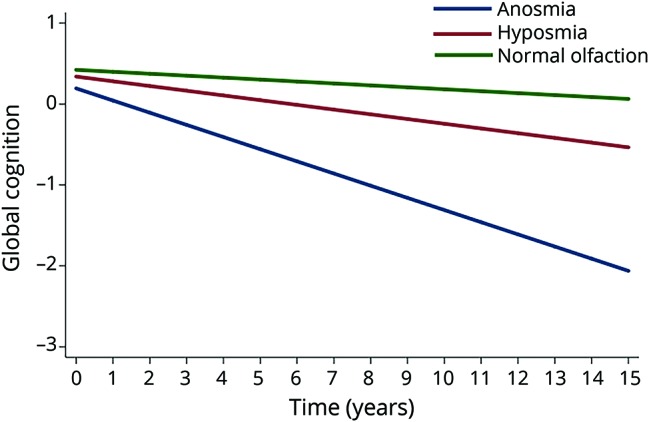
Predicted trajectory of global cognitive decline by olfaction categories Lines represent β coefficients from linear mixed-effects model adjusted for age, sex, education, practice effects, and *APOE* ε4 allele, with normal olfaction as reference group. Green line: normal olfaction (B-SIT score men 10.25–12, women 10.5–12); red line: hyposmia (B-SIT score men 6–10, women 6–10.25); and blue line: anosmia (B-SIT <6). B-SIT = Brief Smell Identification Test.

After adjustment for multiple confounders, higher B-SIT score (continuous) was associated with better baseline global cognitive function and a slower rate of cognitive decline over time after multiadjustment (table 2). Participants with anosmia had lower global cognition than those with normal olfactory function at baseline (table 2 and figure 2). Over the follow-up time, olfactory impairment, including hyposmia and anosmia, was associated with faster global cognitive decline than normal olfactory function in basic-adjusted (age, sex, and education) and multiadjusted (additionally adjusted for *APOE* ε4 and practice effects) mixed-effects models (table 2 and figure 2). Moreover, participants with olfactory impairment had a faster decline in episodic memory, visuospatial ability, perceptual speed, and semantic memory than those with normal function. Participants with anosmia additionally had a faster decline in working memory compared to those with normal function (table 2). Further adjustment for vascular risk factors, vascular diseases, dependency in activities of daily living, diabetes, and stroke showed no material alterations on the given associations; therefore, the estimates from the previous more parsimonious models are reported.

**Table 2:**
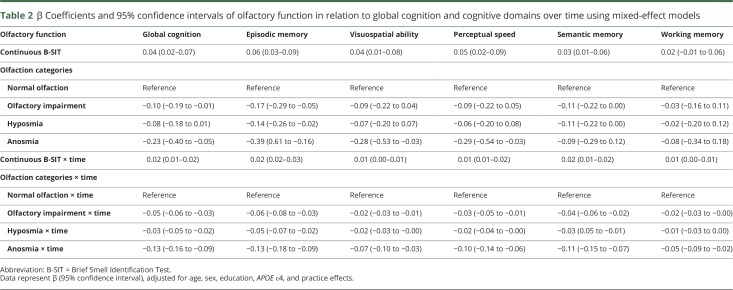
β Coefficients and 95% confidence intervals of olfactory function in relation to global cognition and cognitive domains over time using mixed-effect models

In stratified analysis by *APOE* ε4, the association between olfactory impairment and cognitive decline over time was present in both *APOE* ε4 carries (β = −0.07, 95% confidence interval [CI] −0.12 to −0.02) and ε4 noncarriers (β = −0.04, 95% CI −0.06 to −0.02). There was no interaction between olfactory impairment and *APOE* ε4 on cognitive decline (β = −0.03, 95% CI −0.07 to 0.02, *p* = 0.224).

### Relation between olfactory function and cross-sectional regional brain volumes

**Figure 3:**
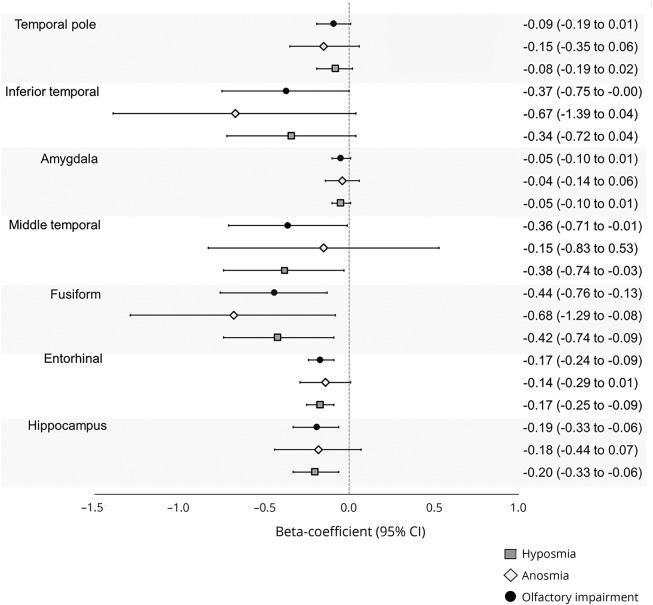
β Coefficients and 95% CIs of olfactory function in relation to regional brain volumes from linear regression (adjusted for age, sex, education, *APOE* ε4, baseline global cognition, and scanning site) Olfaction categories were defined based on baseline Brief Smell Identification Test scores as follows: anosmia (score <6), hyposmia (6–10 men, women 6–10.25), and normal olfaction (10.25–12 men, women 10.5–12). Volumes are expressed as tenths of percentages of intracranial volume. CI = confidence interval.

In linear regression analysis, a higher B-SIT score was associated with greater volumes of the hippocampus, entorhinal cortex, amygdala, fusiform gyrus, temporal pole, and inferior temporal cortex (table 3). Furthermore, participants with olfactory impairment had lower volumes in the hippocampus, entorhinal cortex, middle temporal cortex, and fusiform gyrus compared to those with normal olfactory function (figure 3). There was no interaction between *APOE* ε4 and olfactory categories on volumes of the hippocampus, entorhinal cortex, fusiform gyrus, and middle temporal cortex (data not shown).

**Table 3:**
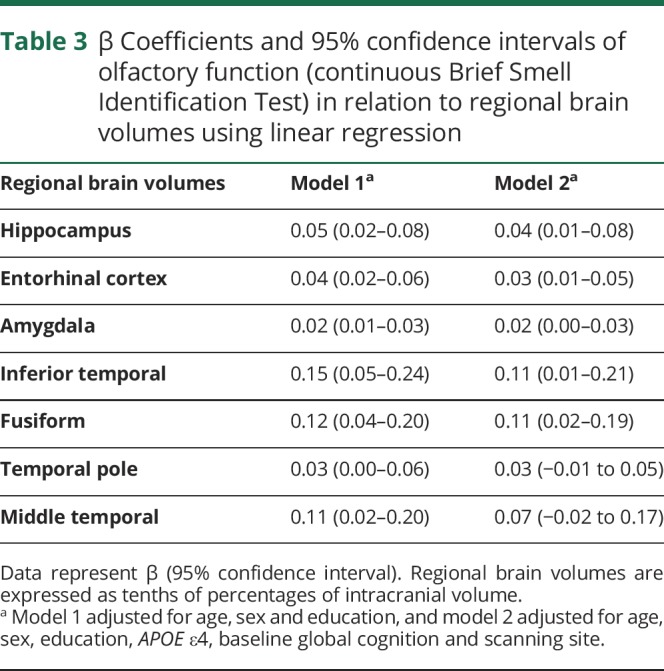
β Coefficients and 95% confidence intervals of olfactory function (continuous Brief Smell Identification Test) in relation to regional brain volumes using linear regression

### Supplementary analysis

We repeated the analyses by excluding incident dementia cases during the follow-up (n = 66). The results were similar to those from the initial analysis, i.e., olfactory impairment remained associated with a steeper global cognitive decline. However, of the 5 cognitive domains, only episodic memory remained associated with olfactory impairment (β = −0.02, 95% CI −0.04 to −0.01). In the MRI analyses, the association between olfactory impairment and volume in the hippocampus, entorhinal cortex, fusiform gyrus, and middle temporal cortex remained significant (data not shown). In addition, we repeated the analyses excluding participants with incomplete odor identification scores (n = 12) and the results were not materially altered.

## Discussion

In this community-based prospective study of dementia-free older adults, we found that olfactory impairment was associated with faster cognitive decline and lower volume in the fusiform gyrus and the middle temporal cortex, including the hippocampus and entorhinal cortex, suggesting that olfactory impairment could be a predictor for subsequent cognitive decline and an indicator of neurodegeneration in the brain.

We previously reported that poorer olfactory performance was associated with faster cognitive decline and AD pathology.^3,17^ In this study, we extended our results to 15 years of follow-up and examined its cross-sectional relation to structural brain MRI measures. A few longitudinal population-based studies have found that poorer olfactory performance is associated with cognitive decline^7–11^; however, most of these studies had short follow-up time. One prospective study with up to 16 years of follow-up found no evidence of an association between odor identification performance and cognitive decline.^12^ These studies have not ruled out the possibility of underlying dementia pathology driving the associations between olfactory impairment and cognitive decline. It has been suggested that the mixed findings are related to the failure to take into account the effects of preclinical dementia,^26^ as the influence of dementia pathology on cognition may begin several years before an established clinical diagnosis.^27^ One study took into account preclinical dementia (dementia diagnosed up to 5 years after baseline),^9^ showing that the pattern of global cognitive decline related to olfactory deficits was similar before and after excluding dementia cases.

In the present study, we found that olfactory impairment was associated with an accelerated cognitive decline over 15 years, which persisted after excluding all incident dementia cases over the follow-up time. Moreover, previous work from the authors has shown a faster decline in perceptual speed and episodic memory. In the current study with longer follow-up time, we observed that impaired olfactory function was additionally associated with visuospatial memory and semantic memory. However, after removing incident dementia cases, only the association with episodic memory remained significant. This suggests that impaired olfactory function could be a predictor of subsequent cognitive impairment and dementia, rather than a marker of already present cognitive impairment.

Atrophy in the primary olfactory cortex (entorhinal cortex and amygdala) has been found in previous studies in young adults with anosmia and hyposmia.^28^ Atrophy in the primary olfactory cortex has also been reported in cognitively normal older adults with olfactory impairment^14,29^ as well as lower activity in this area with olfactory tasks.^30^ While trends have been observed of lower hippocampal volume in individuals with anosmia, olfactory impairment has not traditionally been associated with hippocampal volumetric differences in young adults.^23^ In accordance with these studies, we found that olfactory impairment was associated with lower volume in the fusiform gyrus and the middle temporal cortex (including the hippocampus and entorhinal cortex). The discrepant findings regarding the parahippocampus, orbitofrontal cortex, and precentral cortex may be related to differences in sample size, neuroimaging techniques, and type of olfactory assessment. However, taken together, these findings suggest that olfactory impairment is associated with AD signature areas in dementia-free older adults.

By contrast, it has been suggested that olfactory impairment in old age may be a reflection of neuropathology specific to aging processes rather than AD. We previously found that tangle density within areas of the central olfactory system (i.e., entorhinal cortex, CA1-subiculum) was strongly related to odor identification test scores, whereas tangle density in areas outside the system was not, indicating that neurofibrillar pathology is a contributing factor of impairment in odor identification in old age.^31^ Moreover, hippocampal atrophy has most often been used as a neurodegenerative marker of AD; however, it is not specific for this disease and could be an indicator of neurodegeneration caused by other aging-related processes, including tauopathy.^32^ This is indeed also the case for the amygdala, in which age-related reductions in volume have frequently been reported.^33,34^

In our study of dementia-free participants, we found total medial temporal volume to be associated with olfactory performance. This is consistent with a postmortem brain autopsy study, which reported that a measure of neuritic plaques and neurofibrillary tangles in the medial temporal lobe explained 12% of the variance in odor identification performance while alive.^31^ Furthermore, in neuroimaging studies of older people with AD dementia, olfactory performance has been associated with changes in volume and metabolism of the medial temporal cortex.^35,36^ Because the medial temporal lobe includes structures such as the hippocampus that are vital to memory processing,^37^ this structure may be an important link between age-related olfactory impairment and accelerated cognitive decline.

Previous studies reported a faster decline in cognitive performance only in *APOE* ε4 carriers.^38,39^ However, controlling for prediagnostic dementia, some studies showed a modifying effect of the ε4 allele on cognitive function,^40^ while others did not.^41^ Nevertheless, the magnitude of influence of the ε4 allele on cognition in nondemented populations is typically very small, compared to its influence on AD processes. In our study, stratifying by carriers and noncarriers of the *APOE* ε4 allele showed no difference in the rate of cognitive decline. Moreover, we did not find an interaction between *APOE* ε4 allele status and any of the regional brain volumes. This suggests that the associations of olfactory impairment with accelerated cognitive decline and volumetric differences were independent of *APOE* ε4.

The strengths of this study include the assessment of cognitive function in multiple functional domains with previously established composite measures, which enhanced our ability to identify an association with global cognition, and the repeated yearly follow-up of cognition over a relatively long follow-up time. Moreover, we removed incident dementia cases to address the possibility that the observed association between olfactory impairment and cognitive decline could be driven by underlying dementia. Finally, the very high follow-up of survivors increases the internal validity of associations. However, this study has several limitations. First, our study included only cross-sectional MRI data. Conversely, it is known that brain changes occur before cognitive decline and nearly 15 years before the clinical symptoms of dementia.^42^ Therefore, it is likely the brain abnormalities may underlie the cognitive decline. For the purpose of investigating the mechanisms underlying the relation between olfactory impairment and cognitive decline, future studies should investigate these relationships prospectively to confirm the associations observed in this study. Second, while we used a psychometrically established measure of odor identification, it is a brief form that may be less discriminative, and could have underestimated our results. Moreover, we focused exclusively on odor identification; the association of regional brain volumes with other olfactory functions remains to be investigated. However, a recent meta-analysis suggested that of several olfactory measures, odor identification is one of the most suitable for inclusion in a set of biomarkers to identify subclinical dementia disorder, particularly in combination with neuropsychological assessment and neuroimaging.^43^ Third, the participants were volunteers who were not randomly selected from the community, and were generally well educated and had scored high on cognitive tests. This may have affected the magnitude of our results toward an underestimation. Furthermore, these findings might only be generalizable to similar cohorts and this limitation precludes the generalization of the findings to the general population. Fourth, we were unable to identify and exclude people with a history of allergies, nasal conditions, or diseases that could reduce olfactory function if present.

In this sample of dementia-free older adults, we report a longitudinal association between worse scores on baseline odor identification testing and cognitive decline, and a cross-sectional association between odor identification and volumes in structures of the medial temporal lobe as well as the fusiform gyrus. Future research should further investigate the potential for odor identification tests to serve as cost-effective screening tools for accelerated cognitive decline that may progress to dementia.

## Glossary

AD: Alzheimer disease
B-SIT: Brief Smell Identification Test
CI: confidence interval
MAP: Rush Memory and Aging Project
